# Recent progress in the imaging of c‐Met aberrant cancers with positron emission tomography

**DOI:** 10.1002/med.21885

**Published:** 2022-03-16

**Authors:** Giuseppe Floresta, Vincenzo Abbate

**Affiliations:** ^1^ Department of Analytical, Environmental and Forensic Sciences Institute of Pharmaceutical Sciences, King's College London London UK

**Keywords:** cancer imaging, c‐Met, hepatocyte growth factor, HGF, molecular imaging, PET, targeted molecular probe

## Abstract

Tyrosine‐protein kinase Met—also known as c‐Met or HGFR—is a membrane receptor protein with associated tyrosine kinase activity physiologically stimulated by its natural ligand, the hepatocyte growth factor (HGF), and is involved in different ways in cancer progression and tumourigenesis. Targeting c‐Met with pharmaceuticals has been preclinically proved to have significant benefits for cancer treatment. Recently, evaluating the protein status during and before c‐Met targeted therapy has been shown of relevant importance by different studies, demonstrating that there is a correlation between the status (e.g., aberrant activation and overexpression) of the HGFR with therapy response and clinical prognosis. Currently, clinical imaging based on positron emission tomography (PET) appears as one of the most promising tools for the in vivo real‐time scanning of irregular alterations of the tyrosine‐protein kinase Met and for the diagnosis of c‐Met related cancers. In this study, we review the recent progress in the imaging of c‐Met aberrant cancers with PET. Particular attention is directed on the development of PET probes with a range of different sizes (HGF, antibodies, anticalines, peptides, and small molecules), and radiolabeled with different radionuclides. The goal of this review is to report all the preclinical imaging studies based on PET imaging reported until now for in vivo diagnosis of c‐Met in oncology to support the design of novel and more effective PET probes for in vivo evaluation of c‐Met.

## 1 INTRODUCTION

**Figure 1:**
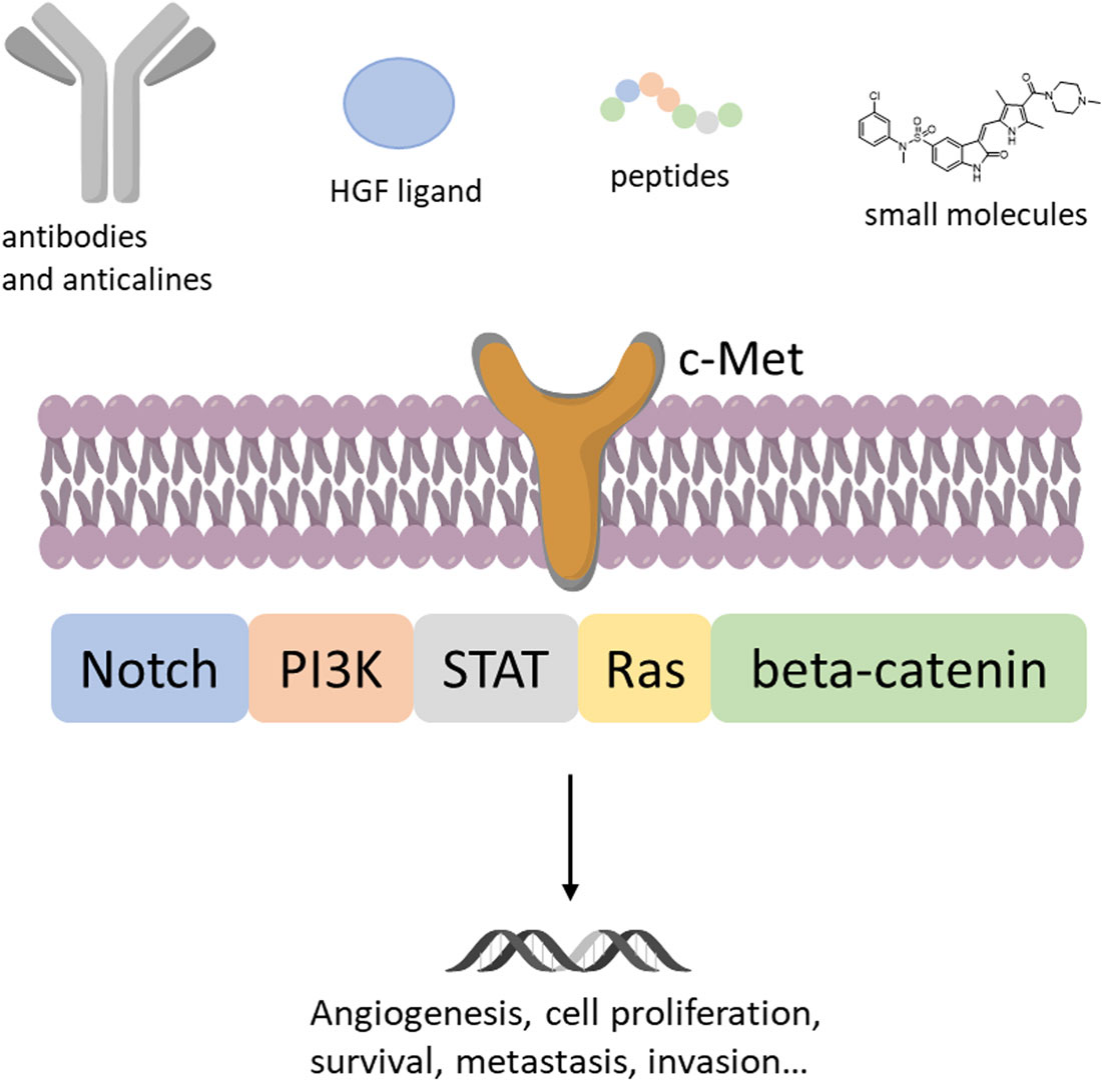
c‐Met/HGF signaling pathway and different classes of PET probes [Color figure can be viewed at wileyonlinelibrary.com]

c‐Met is a membrane receptor protein with tyrosine kinase activity activated by its physiological ligand, namely the hepatocyte growth factor (HGF). This protein, once activated, stimulates a range of intracellular signaling pathways such as those related to proliferation, motility, and invasion/migration of cancer cells.^1^ Unfortunately, this receptor's signaling is aberrantly activated and involved in the initiation and metastatic invasion of several tumor types, including colorectal cancer, prostate, gastric and gastroesophageal cancer, ovarian, renal, lung, cervical, breast, pancreatic cancers, and melanoma.^2^, ^3^, ^4^, ^5^, ^6^ According to the ECIS (European Cancer Information System) cancer has a major impact on society worldwide. The three most common new cases of cancer are breast, colon/rectum, and lung/bronchus for females, and prostate, colon/rectum, and lung/bronchus for males. These types of cancer are also the ones that lead each year to the highest number of deaths. Notably, a significant number of human tumor types, including those already cited, often present the c‐Met receptor overexpressed.^7^ Considering only these four types of cancer, each year almost 7 million new cases worldwide are diagnosed (2 million lung cancer, 1.3 million prostate cancer, over 2 million breast cancer, and 1.8 million colon/rectum cancer); among them, 372,435 new cases of lung cancer, 388,278 of prostate cancer, 415,977 of breast cancer and 388,181 of colon/rectum cancer were reported in Europe in 2018.^8^ In these cancers the overexpression of c‐Met is well documented, for example, 35%–72% for lung adenocarcinoma, 38% for lung squamous cell carcinoma, 23% for hormone‐refractory prostate cancer, 72% for prostate cancer bone metastases, up to 80% for breast cancer, and 10%–75% for colon/rectum cancer,^9^, ^10^, ^11^, ^12^ resulting in a very large target clinical population per year of millions of potential patients worldwide that could benefit from novel c‐Met imaging agents. Moreover, drug resistance, increased metastasis, and poor clinical outcome are unfortunately associated with this receptor overexpression.^13^, ^14^ All of these characteristics indicate that this protein receptor is a key player in several stages of the pathology. Therefore, the real‐time investigation of the expression of c‐Met with positron emission tomography (PET) is likely to assist in the diagnosis and the observation of response to therapy.^15^, ^16^ Particularly, small molecules inhibitors of c‐Met tyrosine kinases activity and antibodies‐based pharmaceuticals with anti‐c‐Met activity have recently shown promising results in the clinical management of c‐Met aberrant cancers. Crizotinib and cabozantinib (both small molecules inhibitors of the protein tyrosine kinase activity) were the two first c‐Met‐inhibitors approved by the US FDA. Specifically, crizotinib was the first approved (2011) for the management of advanced or metastatic non‐small cell lung cancer (NSCLC), and cabozantinib was approved later (2012) for medullary thyroid cancer.^17^ The imaging of the expression of this receptor in real time has the prospect to assist the clinical assessment of c‐Met‐targeted therapies by acceleration in the selection of patients and in monitoring the anti‐c‐Met therapies based on inhibitors of the protein.^18^, ^19^ Improved diagnostic methods for the identification of patients suitable for c‐Met targeted treatment are of primary importance to improve the outcome of c‐Met aberrant cancers in the clinic. Nowadays, patient selection is normally conducted by fluorescent in situ hybridization or immunohistochemistry. Both methodologies can yield quantitative information about c‐Met expression, but they have critical limitations: they are not able to reflect the c‐Met expression variation over time, they cannot deal with the receptor heterogeneity in different tumor sites, biopsies cannot be conducted on inaccessible sites, and they provide only a small sample of heterogeneous tissue within a single tumor, in addition to the fact that recurring biopsies can be hurtful and difficult for the patient. Considering this scenario, molecular imaging based on PET can defeat these drawbacks because it offers high sensitivity real‐time detection of biomolecular events. Many imaging tracers based on different detection techniques (gamma camera,^20^, ^21^, ^22^, ^23^, ^24^, ^25^, ^26^ magnetic resonance imaging [MRI],^27^, ^28^, ^29^, ^30^ and fluorescence^31^, ^32^) have been reported for c‐Met.^33^ Also, several radiolabeled antibodies, peptides, or small molecules against this protein have been used for in vivo cancer localization; however, there has not been any clinical translation of PET tracers for c‐Met produced to date. In this study, we review the latest progress in the imaging of c‐Met aberrant cancers with PET. Particular attention is directed on the development of PET imaging probes with a broad range of molecular sizes (HGF, antibodies, anticalines, peptides, and small molecules; Figure 1, Table 1) and incorporating different radionuclides. The goal of this review is to report all the in vivo real‐time PET‐based imaging studies reported until now for monitoring of c‐Met in cancer, to support the design of novel and more effective PET probes for in vivo evaluation of the protein.

**Table 1:** PET imaging agents developed for c‐Met imaging in cancer

Name	Nature	Tumor model	Control	References
^64^Cu‐NOTA‐rh‐HGF	HGF ligand	U87‐MG	MDA‐MB‐231	^34^
^89^Zr‐DN30	Monoclonal antibody	GTL‐16	FaDu	^35^
^89^Zr‐onartuzumab	Monoclonal antibody	MKN‐45	U87‐MG	^36^
^76^Br‐onartuzumab	Monoclonal antibody	MKN‐45	U87‐MG	^36^
^89^Zr‐1E2‐Alb8	aHGF‐Nanobody	U87‐MG	–	^37^
^89^Zr‐6E10‐Alb8	aHGF‐Nanobody	U87‐MG	–	^37^
^89^Zr‐DFO‐H2 cys‐diabody	Single‐chain variable fragment	Hcc827‐GR6	Hcc827	^38^
^89^Zr‐PRS‐110	Anticalin	H441, U87‐MG	A2780	^39^
^89^Zr‐DFO‐AMG102	HGF monoclonal antibody	U87‐MG	MKN‐45	^40^
^89^Zr‐onartuzumab	Monoclonal antibody	HCC827ErlRes	HCC827	^19^
^68^GaGaHBED‐CC‐azepin‐MetMAb	Monoclonal antibody	MKN‐45	PC‐3	^41^
^89^Zr‐onartuzumab	Monoclonal antibody	BxPC3, Capan2, Suit2	MIA PaCa‐2	^42^
^89^ZrDFO‐azepin‐onartuzumab	Monoclonal antibody	MKN‐45	–	^43^
^89^ZrDFO‐Bn‐NCS‐onartuzumab	Monoclonal antibody	MKN‐45	–	^43^
^89^ZrDFO‐amivantamab	Bispecific antibody	MDA‐MB‐468, MDA‐MB‐231	MDA‐MB‐253	^44^
^18^F‐AH113804	Peptide	HCC1954	HT‐29, U87	^45^
^18^F‐FP‐Met‐pep1	Peptide	UM‐SCC‐22B	‐	^46^
^64^Cu‐HiP‐8‐PEG11	Peptide	PC‐9 (HGF+)	PC‐9 (HGF−)	^47^
^68^Ga‑EMP‑100	Peptide	Metastatic renal cell carcinoma^a^	–	^48^
^11^C‐SU11274	Small molecule	H1975	H520	^49^
^18^F‐cabozantinib	Small molecule	–	–	^50^
^18^F‐TPC	Small molecule	H1993	A549	^51^

Abbreviations: HGF, hepatocyte growth factor; PET, positron emission tomography.

^a^ In‑human biodistribution and imaging study.

It is reasonable to hypothesize that, in future clinical settings, noninvasive imaging with PET and using these new tracers will support the diagnosis of c‐Met overexpressed cancers and the selection/evaluation (responding and nonresponding) of patients for c‐Met–targeting drugs.

## 2 PET PROBES BASED ON THE HGF LIGAND

Due to the specificity and high binding affinity of the natural ligand for c‐Met, the HGF structure was initially selected for the first imaging studies of the receptor (by MRI).^52^ The only example of a PET probe based on the HGF was reported by Luo et al.^34^ The reported product was a recombinant human HGF functionalized with the NOTA chelating moiety then labeled with ^64^Cu for c‐Met‐targeted molecular imaging.^34^ To synthesize this tracer,

2‐S‐(4‐Isothiocyanatobenzyl)−1,4,7‐triazacyclononane‐1,4,7‐triacetic acid (p‐SCNBn‐NOTA) was covalently bonded to rh‐HGF. The resulting product ^64^Cu‐NOTA‐rh‐HGF was assessed by flow cytometry using human glioblastoma cell lines (U87‐MG) with moderate c‐Met expression and human breast cancer (MDA‐MB‐231) with lower (compared to U87‐MG) c‐Met expression, confirming the specific binding of the labeled HGF to c‐Met overexpressing cells. U87‐MG xenografts mice were used for in vivo experiments, revealing rapid tumor absorption of ^64^Cu‐NOTA‐rh‐HGF that was distinctly visible at 30 min postinjection with a peak at 9 h (6.7 ± 1.8% ID/g, percentage injected dose per gram). Contrarily, a lower absorption was reported for MDA‐MB‐231 mice xenografts. These results are consistent with the different expressions of the protein in the two cell lines used in the xenografts. Heating of the ^64^Cu‐NOTA‐rh‐HGF resulted in denaturation of the protein and the denatured product, termed ^64^Cu‐NOTAdnrh‐HGF, had notably reduced uptake in U87‐MG bearing mice than the unheated ^64^Cu‐NOTA‐rh‐HGF. The specificity of ^64^Cu‐NOTA‐rh‐HGF was also established by the fact that its uptake in all other main organs was comparable between the natural and the denatured form of the tracer. As expected, the liver and kidney uptake values were similar between xenografts injected with the natural and the denatured form, because these organs represent the clearance tissues for a molecule of this size (~70 kDa). This study demonstrates the potential application of imaging agents based on the structure of the endogenous HGF for c‐Met imaging. Limitation of this approach, precluding its clinical translation, could be due to the concomitant presence of endogenous HGF that will induce the classical biological effects of the HGF, that is, cell proliferation/survival, and could also promote tumor growth. Moreover, the higher concentration of the endogenous ligand could preclude the interaction of the radiolabeled ligand with c‐Met with subsequent reduction of the contrast ratio of the tumor PET images.^53^

## 3 PET PROBES BASED ON ANTIBODIES AND ANTICALINES

Various monoclonal antibodies (mAbs) and anticalines targeting c‐Met or its ligand (HGF), for example, DN30, rilotumumab, onartuzumab, and PRS‐100, have been recently tested in both preclinical and clinical trials, with encouraging results for the management of cancer.^54^, ^55^, ^56^ Therefore these classes of compounds have also started to attract the interest in nuclear medicine pursuing imaging agents for the membrane‐targeted receptor.

In 2008, DN30 (a mAb against c‐Met) was first radiolabeled and used in in vivo PET imaging experiments by Perk et al.^35^ with ^89^Zr for detecting c‐Met receptor. The radioactive isotope of zirconium was combined to the mAb through N‐Succinyldesferrioxamine‐DN30 (N‐sucDf‐DN30, a desferrioxamine‐based chelating moiety). Biodistribution studies were carried out with different xenografted mice models, with high and low expression of the receptor (high: GTL‐16, human gastric cancer; low: FaDu, human head‐and‐neck cancer). The absorption of ^89^Zr‐N‐sucDf‐DN30 in the low expressing c‐Met cell line was substantially lower than that in GTL‐16, reflecting the different c‐Met concentrations in the two cell lines; precisely, at 3 days postinjection 7.8 ± 1.2% ID/g was detected in FaDu and 18.1 ± 4.5% ID/g in GTL‐16 xenografts. In line with these results, human gastric cancer as small as 11 mg lesions were sharply visualized.

Jagoda et al.^36^ used ^76^Br and ^89^Zr for the radiolabeling of onartuzumab in 2012 and tested the labeled monoclonal antibody against c‐Met overexpressing human cancer cell lines and xenografted mouse models. Onartuzumab was labeled with ^89^Zr using desferrioxamine as chelating moiety while ^76^Br was directly covalently bonded. Biodistribution studies were conducted in MKN‐45 (human gastric carcinoma, high c‐Met expression) and U87‐MG tumors (moderate level c‐Met expression). Uptake of both the studied radioisotopes was consistent with the targeted receptor expression levels in the two different studied cell lines, namely uptake in MKN‐45 was higher than U87‐MG. The distribution in nontarget tissues was similar for the two different labeled onartuzumab in all tumor models. Competitive blocking by unlabeled onartuzumab in the MKN‐45 xenografts resulted in 3.8‐fold decreased uptake of the tracer. Between the two labeled compounds, the ^76^Br‐onartuzumab showed lower retention in the tumor tissue with a faster clearance. MKN‐45 xenografts were detected by micro PET imaging at 18–24 h postinjection with ^76^Br‐onartuzumab (with an optimum image quality at 24 h), whereas images generated with ^89^Zr‐onartuzumab continued to improve in quality over 5‐day postinjection. The accumulation of ^89^Zr‐onartuzumab in the gastrointestinal tract, lungs, heart, blood, and muscle decreased gradually over time, with more than 50% decrement from 18 h to 5 days, whereas uptake in kidney and liver increased progressively over the studied period, remarking renal and hepatobiliary clearance. Of note, a high bone absorption up to 7% ID/g was also measured at 5‐day postinjection, possibly due to free zirconium released from the tracer.

**Figure 2:**
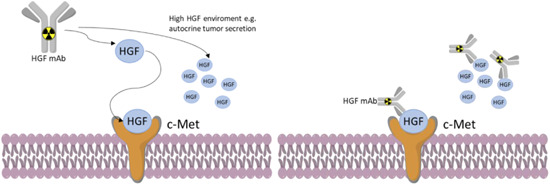
Depiction of anti‐HGF antibodies labeled with radioactive metal for use in PET imaging of HGF/c‐Met positive cancers. HGF, hepatocyte growth factor; PET, positron emission tomography [Color figure can be viewed at wileyonlinelibrary.com]

A different approach to target the HGF/c‐Met pathway was investigated by van Dongen et al. in 2012, where two anti‐HGF (αHGF) nanobodies, named 1E2 and 6E10, were developed to investigate HGF in vivo concentration by PET imaging (Figure 2).^37^ To extend their serum half‐life the two nanobodies were engineered by coupling them to an albumin‐binding nanobody unit (Alb8) to obtain 1E2‐Alb8 and 6E10‐Alb8, then were radiolabeled with ^89^Zr. U87‐MG glioblastoma xenografts were used for biodistribution studies. Both the tracers (1E2‐Alb8 and 6E10‐Alb8) showed decreasing blood levels over 7‐day postinjection while tumor uptake levels remained relatively stable during the same period, making them suitable probes for PET scanning. The absorption in other tissues was minor than in tumor tissues, aside from kidneys that control the clearance of these proteins. In addition, the two nanobodies were evaluated for their therapeutic effect, due to their anti‐HGF activity, resulting in tumor growth inhibition after treatment with 100 mg intraperitoneal injections, 3 injections per week over 5 weeks.

scFv‐cysdimers H2, a human single‐chain variable fragments‐cys‐diabody was radiolabeled with ^89^Zr and studied in xenografts mouse models by Li et al.^38^ The cys‐diabody was labeled with the radiometal using a deferoxamine‐maleimide covalently bonded to the antibody.^89^Zr‐DFO‐H2 cys‐diabody uptake was studied in Hcc827‐GR6 (human NSCLC with high c‐Met expression resistant to gefitinib) and Hcc827 tumors (human NSCLC with low c‐Met expression). The uptake was greater in Hcc827‐GR6 cell lines at 4 and 20 h postinjection in accordance with the c‐Met cellular expression levels. Hcc827‐GR6 showed an uptake of 1.8 ± 0.2% ID/g, compared to Hcc827 with only 0.65 ± 0.15% ID/g, as evaluated by ex vivo biodistribution studies.

Due to their small size (less than 20 kDa), anticalins could represent a valid alternative to mAb (150 kDa) with higher tumor uptake and tissue penetration and hence potentially improved characteristics for PET tracers.^57^ PRS‐110, a specific anticalin for c‐Met, was engineered by Terwisscha van Scheltinga et al.^39^ PRS‐100 half‐life was prolonged by conjugating the anticalin to a branched 40‐kDa polyethyleneglycol, and the binding affinity for the c‐Met receptor was measured as 0.6 nmol/L. The branched PEG moiety of PRS‐100 was reacted with deferoxamine‐p‐SCN and afterward labeled with ^89^Zr. PET scans were done for mice bearing H441 (high c‐Met expression), U87‐MG (moderate c‐Met expression), and A2780 (low c‐Met expression) cell lines. Specific tumor uptake was clearly seen for ^89^Zr‐PRS‐110 in both H441 and U87‐MG xenografts whereas the A2780 mice revealed a lower absorption compared to the control (^89^Zr‐Tlc‐PEG, non‐c‐Met binding). PET scans of H441 xenografts mice resulted in a peak uptake at 48 h post ^89^Zr‐PRS‐110 injection that was substantially higher than for ^89^Zr‐Tlc‐PEG, in agreement with the ex vivo experiments of biodistribution (5.9% ID/g for ^89^ZrPRS‐110 vs. 3.9% ID/g for ^89^Zr‐Tlc‐PEG). In PET scans of A2780 bearing mice, both tracers resulted in similar uptake, in agreement with c‐Met expression levels (1.7% and 2.5% ID/g, respectively). Biodistribution data showed that the uptake in other nontumour organs was comparable except for lungs; this could be explained by possible metastasis of H441 tumors.

A similar approach to van Dongen^37^ was developed by Lewis et al. in 2017, where AMG102 (rilotumumab, antibody for HGF) was engineered for its use as ^89^Zr PET imaging agent.^40^ AMG102 was covalently bonded to the chelator p‐SCN‐Bn‐DFO and then labeled with the radiometal, and the binding affinity to the HGF was studied using an immunosorbent (ELISA, enzyme‐linked immunosorbent assay) binding assay. Mice xenografts of MKN45 (HGF‐negative, c‐Met‐positive) and U87MG (HGF‐positive, c‐Met‐positive) were employed for animal PET imaging, ex vivo experiments, immunohistochemistry, and biodistribution studies. Four patient‐derived xenografts of gastric cancer (c‐Met‐positive, HGF unknown) were also used in the same experiments. As expected from the c‐Met/HGF concentration in the different cell lines, the uptake of the labeled product ^89^Zr‐DFO‐AMG102 at 120 h postadministration was high for U87MG mice (36.8 ± 7.8% ID/g), but low (only 5.0 ± 1.3% ID/g) for MKN45. Uptake experiments in the four patient‐derived gastric cancer xenograft models suggested a low concentration of HGF (4%–7% ID/g). The low levels of the HGF in these tumors were then confirmed by ex vivo ELISA assays. This result shows how ^89^Zr‐DFO‐AMG102 could be useful to real time and noninvasively diagnosis of local HGF levels in tumors.

Another ^89^Zr‐labeled onartuzumab (c‐Met antibody) was studied for its application as PET probe for discriminating the fluctuation in c‐Met expression induced by erlotinib (tyrosine kinase inhibitor) and luminespib (heat shock protein‐90, Hps90 inhibitor) in human NSCLC xenografts by Pool et al.^19^ These experiments delineate the possibility of in vivo imaging of c‐Met receptor with ^89^Zr‐onartuzumab by detecting the protein upregulation due to erlotinib induced resistance, as well as downregulation of the receptor followed by HSP90‐directed therapy in human NSCLC xenograft‐bearing mice. Onartuzumab was conjugated to tetrafluorophenol‐N‐succinyldesferal‐Fe^3+^ (Df) and then studied in animal models. Mice bearing HCC827 (human NSCLC) and HCC827ErlRes (erlotinib resistant human NSCLC with c‐Met upregulation) tumors went through ^89^Zr‐onartuzumab PET scans. The pharmacological treatment of the cells with erlotinib or luminespib resulted in 213 ± 44% c‐Met upregulation in HCC827ErlRes tumors compared to HCC827 tumors, whereas the receptor was downregulated (minus 69 ± 9%) in HCC827 cells following the application of the Hps90 inhibitor. PET scans resulted in a 24% higher ^89^Zr‐onartuzumab absorption in HCC827ErlRes than in HCC827 tumors, moreover, biodistribution data showed that the uptake of the studied probe was higher in HCC827ErlRes (38.1 ± 8.4% ID/g) than in HCC827 cell lines (30.2 ± 3.5% ID/g) confirming that ^89^Zr‐onartuzumab used in PET scan productively contradistinguish modification in c‐Met protein levels.

**Figure 3:**
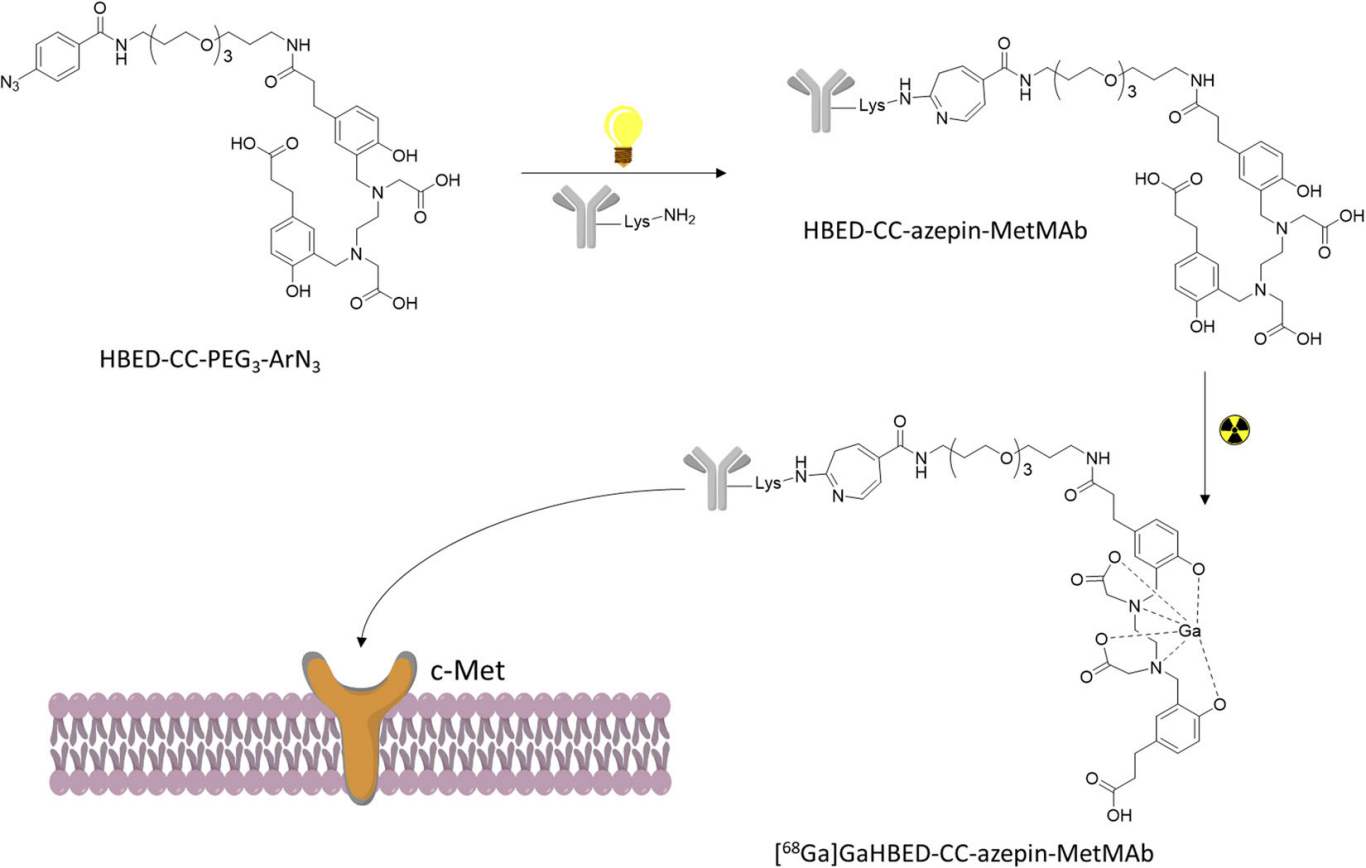
Reaction depiction the photochemical conjugation and radiolabeling to yield [^68^Ga]GaHBEDCC‐azepin‐MetMAb [Color figure can be viewed at wileyonlinelibrary.com]

An alternative approach for the functionalization of onartuzumab was recently reported by Holland et al. who photoconjugated the anti‐c‐Met antibody with a chelating moiety then labeled the conjugate with gallium‐68.^41^ The chosen gallium chelating moiety was an acyclic photoactive molecule named HBED‐CC‐PEG3. A derivative of the HBED‐CC‐PEG3 (HBED‐CC‐PEG3‐ArN3) was produced and conjugated to onartuzumab as shown in Figure 3. The photoconjugation relied on an already reported mechanism where the nucleophilic attack on the photoinduced ketenimine intermediate (from the chelating moiety HBED‐CC‐PEG3‐ArN3) by a primary amine (from the protein) yields the production of a covalent bond between the two species through the formation of an azepine ring (Figure 3).^58^ The photochemical reaction gave the conjugated HBED‐CC‐azepin‐MetMAb with an 18.5% conversion. The stability of the radiolabeled compounds was studied in incubated human serum albumin and only a small (less than 5%) amount of radioactivity was lost over 3 h. Cellular binding studies were conducted by measuring the immunoreactive fraction in vitro using two different cell lines; MKN‐45 (high c‐Met levels) and PC‐3 (low to moderate c‐Met levels). PET images were recorded in groups of xenografted mice bearing MKN‐45 and PC‐3 tumors which showed tumor‐associated activities after 6 h of 10.33 ± 1.27% and 3.88 ± 1.27% ID/g, respectively. Finally, the uptake of MKN‐45 was reduced by 55% in competitive blocking experiments. As the authors acknowledge, even if they were able to visualize the tumors in their experiment, the use of a short life radionuclide (^68^Ga *t*_1/2_ = 68 min) with a high molecular weight probe is suboptimal, however, the same HBED‐CC‐azepin‐MetMAb could have other potential other applications with smaller peptides or could be labeled with ^67^Ga (half‐life = 78.3 h) or ^111^In (half‐life = 67.3 h) and exploited for radioimmunotherapy (RIT) or as single‐photon emission computed tomography (SPECT).^59^

In a recent study, the already reported ^89^Zr‐onartuzumab ^36^ was studied for its application in pancreatic ductal adenocarcinoma (PDAC).^42^ This type of cancer unfortunately has limited therapeutic options and tyrosine kinases inhibitors trials gave mixed results, somewhat due to suboptimal patient selection.^89^Zr‐onartuzumab was used to show that in PDAC otherwise resistant to c‐Met kinase activity inhibitors, immuno‐PET can diagnose potential patients for targeted radioligand therapy.^89^Zr‐onartuzumab was evaluated against Suit2, Capan2, BxPC3, and MIA PaCa‐2, all tumors resistant to tyrosine kinases inhibitors, despite overexpression of c‐Met. The viability of the cell lines was decreased in vitro by crizotinib and cabozantinib, whereas the less potent capmatinib showed no tyrosine kinase inhibition in PDAC lines. Selective absorption of ^89^Zr‐labeled DFO‐onartuzumab in xenografts mice injected with PDAC cell lines expressing high levels of c‐Met (Suit2, Capan2, and BxPC3) was observed, but no uptake was observed in xenografts with lower levels of c‐Met (MIA PaCa‐2). Onartuzumab was then labeled with lutetium‐177, a beta‐emitting nuclide. [^177^Lu]Lu‐DTPA‐onartuzumab was subcutaneously administered at 9.25MBq (250 μCi)/20 μg in three injections separated by three days in xenograft mice bearing BxPC3 (high c‐Met expression) or MIA PaCa‐2 (lower c‐Met expression). In both animal models, an important delay in tumor growth and survival benefit was accomplished in comparison to control experiments; the benefit was long‐lasting and generally stronger in the BxPC3 animals treated with the lutetium‐177 labeled radiopharmaceuticals. These results revealed that while the overexpression of the studied protein is not prognosticative of the tyrosine kinase inhibitor response, PET scan can identify overexpression of c‐Met in vivo and anticipate therapeutic response to radioligand treatment.

**Figure 4:**
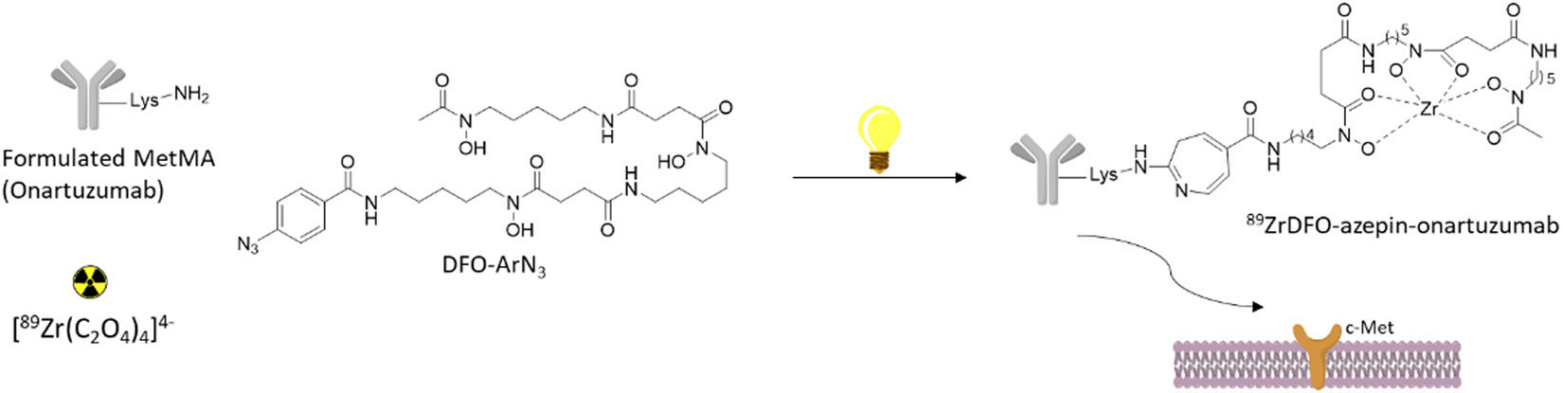
One‐pot multiple component photoradiosynthesis of ^89^ZrDFO‐azepin‐onartuzumab [Color figure can be viewed at wileyonlinelibrary.com]

Another photosynthetic approach for producing another ^89^Zr‐radiolabeled onartuzumab was recently proposed by Holland et al. Differently to the already discussed method,^41^ where the photosynthesis was used to conjugate the chelating moiety for gallium and then the product was radiolabeled in a different step, in this novel approach the conjugation (by photoreaction) and the radiolabeling are made in one‐pot reactions starting from the fully formulated MetMAb (Figure 4).^43^ ^89^ZrDFO‐azepin‐onartuzumab was produced in the same reaction between ^89^Zr‐oxalate, MetMAb and the photoactive chelator DFO‐aryl azide (DFO‐ArN_3_), exploiting the chelating properties of DFO and the formation of an azepine ring between the azido group of the DFO and a lysine of the protein.^89^ZrDFO‐Bn‐NCS‐onartuzumab was also produced in a more classical two‐step way exploiting the same antibody and DFO‐BnNCS. Both compounds were immunoreactive to c‐Met and stable in human serum. MKN‐45 xenograft mice were used for PET imaging and along with biodistribution experiments demonstrate high tumor absorption for both the studied tracers. The uptake was studied at 72 h, in these experiments, the ^89^ZrDFO‐azepin‐onartuzumab tumor and liver uptake peaked at 15.37 ± 5.21%, 6.56 ± 4.03% ID/g, respectively^89^; ZrDFO‐Bn‐NCS‐onartuzumab uptake was 21.38 ± 11.57% ID/g for the tumor and 18.84 ± 6.03% ID/g for the liver, demonstrating again that photoradiosynthesis is a valid option for the production of zirconium‐89 radiolabeled antibodies easily in simple formulation medium. Blocking experiments were also performed and resulted in a significant reduction in tumor uptake of ^89^ZrDFO‐azepin‐onartuzumab to only 6.34 ± 0.47% ID/g.

Recently, Cavaliere et al.^44^ designed a bispecific DFO‐conjugate ^89^Zr antibody, Amivantamab. Amivantamab is a novel antibody with bispecific activity capable of synchronously targeting c‐Met and the epidermal growth factor receptor (EGFR). The bispecific antibody was covalently bonded to desferrioxamine and subsequently radiolabeled to obtain [^89^Zr]ZrDFO‐amivantamab (specific activity of 148 MBq/mg), synthesized with a radiochemical yield higher than 95%. The binding of the labeled conjugate was then studied in vitro and in vivo. Biodistribution studies were performed in MDA‐MB‐468 xenografts mice, differently a panel of triple‐negative breast cancer xenografts mice with various levels of c‐MET and EGFR expression was employed for the PET/CT imaging using the reported zirconium‐89 labeled antibody that, accordingly with c‐Met expression, resulted in higher cellular accumulation of the tracer in MDA‐MB‐231 and MDA‐MB‐468. The imaging findings were also confirmed by the biodistribution studies that showed that the tumor uptake of the radiolabeled tracer in the MDA‐MB‐468 mice was higher than those in the MDA‐MB‐231 and MDA‐MB‐453 xenografts (1.7‐ and 3.5‐fold, respectively). c‐MET and EGFR expression levels were studied by Western blot and radioligand binding studies confirming the order of the proteins expression levels: HCC827 (positive control) > MDA‐MB‐468 > MDA‐MB‐231 > MDA‐MB‐453, demonstrating that the novel amivantamab based tracer showed specificity to cell lines with high level of c‐MET and EGFR. The standard uptake value of the radiolabeled amivantamab in TNBC xenografted was also in agreement with the different receptors expression levels of the studied cells.

In agreement to findings of the above‐reported works, monoclonal antibodies, single‐chain variable fragment diabodies, and anticalins able to target c‐Met, mostly radiolabeled with ^89^Zr, can detect the protein expression in vitro and in vivo in mice models. These promising probes could not only be useful for the diagnosis of c‐Met aberrant cancer but also could enable real‐time monitoring for the selection of patients for c‐Met targeting drugs by the identification of responding and nonresponding patients for c‐Met targeted therapeutics.

## 4 PET PROBES BASED ON PEPTIDES

Compared to the high molecular weight antibody‐based imaging probes, molecular imaging agents with peptide structures present diverse advantages cause of their lower molecular weight. Among them are lower toxicity, higher permeability, favorable pharmacokinetics and tissue distribution, limited immunogenicity, and convenient for controlled chemical modification.^60^ Recently, several c‐Met‐targeted molecular probes (PET, MRI, SPECT, NIR, and Fluorescence) employing c‐Met targeting peptides have been reported allowing the visualization of the receptor expression in vivo with different techniques.^33^ Here, we summarize the recent advantages in peptide PET‐based molecular probes for c‐Met; the sequences of the most promising targeting peptides of other reported probes (not necessarily evaluated via PET but also with other imaging techniques) are illustrated in Table 2. All of these presented sequences could represent the starting point to future PET‐based molecular probes, maintaining the same sequence but varying the radioactive source in the structure, leading to different final probes exploiting different radionuclides that have not been used yet.^63^

**Table 2:** Sequences of the most promising targeting c‐Met imaging peptides

Name	Sequence^a^	Imaging technique	References
GE‐137/^18^F‐AH113804	AGS*C*Y**C**SGPPRFE**C**W*C*YETEGT^a^	Fluorescence/PET	^32^, ^45^
cMBP‐X‐Cy5.5/^99m^Tc‐HYNIC‐cMBP,^125^I‐cMBPs	KSLSRHDHIHHH	Fluorescence/SPECT	^18^, ^20^, ^26^
Met‐pep1	YLFSVHWPPLKA	Scintillation/PET	^21^, ^46^
aML5‐FL	**Ac** ^L^YISWNEFNSPNWRFI**C**G^β^A^b^	Fluorescence	^61^
aMD5‐FL	**Ac** ^D^YRQFNRRTHEVWNLD**C**G^β^A^b^	Fluorescence	^61^
QQT*‐Cy5.5	QQTNWSL	Fluorescence	^62^
HiP‐8	**Ac** ^D^WPLSKWWYSKR**C** b	PET	^47^

Abbreviations: PET, positron emission tomography; SPECT, single‐photon emission computed tomography.

^a^ S–S bond between the bold and the italic AA.

^b^ Thioether linkage between the acetyl and the cysteine in bold.

Based on an already published fluorescent peptide (GE‐137)^32^ that was successfully employed for the identification of colorectal polyps in humans after intravenous administration, Arulappu et al.^45^ developed ^18^F‐AH113804 for c‐Met detection via PET. The peptide was shown to bind c‐Met with high specificity and with a binding affinity of about 2 nM (*K*_d_). The measured levels of radioactivity 1 h postinjection were 2.0 ± 0.2%ID/ml in the blood, 2.9 ± 0.3%ID/ml in liver, and 4.9 ± 0.6%ID/ml in kidney.^18^F‐AH113804 uptake in the tumor site was 1.5 ± 0.2%ID/ml, compared with both muscle 0.8 ± 0.1%ID/ml and contralateral mammary fat pad 0.8 ± 0.1%ID/ml at 60 min after injection.^18^F‐AH113804 was then used in PET imaging to reveal reappearance after surgical excision of HCC1954 human basal‐like breast cancer implanted mice with high c‐Met levels as early as 6 days after the operation, showing the great potential of the probe in PET imaging as a clinical screening after surgical ablation of HCC1954 breast cancer. Moreover, by virtue of the peptide‐like properties (i.e., lower molecular weight than antibodies) ^18^F‐AH113804 (3.2 kDa) permitted high‐contrast image (1 h postinjection), that is, improved sensitivity for cancer detection. A peptide similar to ^18^F‐AH113804 named AH‐113018 was also proposed by Jagoda et al.,^64^ already mentioned in the antibodies paragraph, that was labeled with ^99m^Tc and used for SPECT imaging. The clinical safety of ^18^F‐AH113804 was confirmed by a phase‐1 biodistribution study in healthy adult volunteers.^65^

Met‐pep1, a c‐Met binding peptide,^21^ was modified by Li et al.^46^ for its use in PET imaging. After the synthesis of the linear sequence of the c‐Met‐binding peptide, this was conjugated with ^18^F‐2‐ fluoropropionate resulting in ^18^F‐FP‐Met‐pep1. UM‐SCC‐22B (head and neck human squamous carcinoma with high c‐Met expression) cells were used to measure the internalization, cellular uptake, and efflux of the fluorine‐18 labeled Met‐pep1, while tumor‐bearing nude mice and PET imaging were used to measure the biodistribution and the pharmacokinetics. The uptake of the radiotracer was high in UM‐SCC‐22B mice with excellent PET imaging results. Both the cellular uptake and the internalization of the tracer followed a similar trend displaying a fast increase during the first 15 min, encompassing their concentration peak after 30 min and decreasing at 60 min. Efflux experiments showed that in the first 15 min 47% of the radiotracer was taken out of the cells, after 30 min the efflux rate slowed down and at the end of the incubation the tracer bound to cells was about 36%. PET imaging experiments showed that UM‐SCC‐22B tumors were clearly visible with uptakes of 4.72%, 3.83%, and 3.11% ID/g at 30, 60, and 120 min postinjection, respectively. Coadministration of unlabeled Met‐pep1 was used to confirm the specificity of the tracer that resulted in decreased tumor uptake showing a high specificity. High values of organ absorption were measured in the kidneys (6.15 ± 0.71% ID/g) and in the liver (11.5 ± 0.7% ID/g), but a rapid renal clearance (65% excreted within 2 h after) suggested a mainly renal‐urinary excretion of the tracer. Muscle and other organs (including intestine, stomach, bone marrow, spleen, lung, and heart), had very low uptake during all time points.

Exploiting random nonstandard peptides integrated discovery (RaPID) selection,^66^ Sakai et al. recently identified HiP‐8 (macrocyclic peptide, 1.6 KDa), as a novel functionally active two‐chain HGF (tcHGF)^67^, ^68^ binding peptide that strongly inhibited the interaction of HFG with c‐Met with subnanomolar potency.^47^ Noninvasive screening/visualization and inhibition of HGF/c‐Met was observed after iv administration of HiP‐8 followed by PET imaging in a mouse model. HiP‐8 showed a high binding affinity to HGF with a *K*_d_ of 0.4 nM and a *k*_off_ of 0.4 × 10^−3^ s^−1^ (dissociation rate) was measured by surface plasmon resonance. The reported HGF‐inhibitory activity in cellular assays (as IC_50_) was 8 nM. The water solubility and the pharmacokinetics of the macrocyclic peptide were improved by the inclusion of a polyethylene glycol chain to the C‐terminus. The selectivity of HiP‐8 to HGF was also compared with other similar proteins human growth factors, resulting in a high selectivity for HGF. Murine HGF was also evaluated and HiP‐8‐PEG11 displayed binding to the nonhuman grow factor with 50‐fold less affinity. The use of HiP‐8‐PEG11 was then evaluated for PET scansion of HGF–Met activation in tumors. To achieve this, the molecule was chemically engineered and an ‐N_3_ group was added to a lysine by using a Fmoc‐Lys(N_3_)‐OH, that was subsequently conjugated to the copper chelating ligand DBCO(dibenzocyclooctyne)‐PEG4‐CB‐TE1K1P (TE1K1P is a bifunctional chelator for copper radionuclides).^69^ The obtained product was then labeled with ^64^Cu, resulting in a radiochemical purity ≥95% measured by radio‐HPLC. The tracer was then administered intravenously to SCID mice xenografts with PC‐9 tumors (HGF− and HGF+) showing liver absorption, a fast renal clearance, and tumor accumulation. The tumor uptake reached a peak between 10 and 17 min, the tumor/muscle ratio of radioactivity—contrast index—showed accumulation of the tracer in HGF positive tumors with high selectivity (5.74 tumor/muscle ratio of radioactivity for HGF−, 20.16 tumor/muscle ratio of radioactivity for HGF+), demonstrating that ^64^Cu‐HiP‐8‐PEG11 is an outstanding probe for noninvasive imaging of HGF–Met status in tumors on an animal model by PET imaging. Moreover, due to the optimal characteristics of the c‐Met targeting peptide and to the practicality of chemical modification, HiP‐8 could be exploited for other applications in imaging by conjugation with different chelating units and/or other chemical modifications.

Based on the structure of GE‐137,^70^ ^68^Ga‑EMP‑100 was recently designed and used in a pioneer in‐human imaging and biodistribution study.^48^ ^68^Ga‐EMP‐100 is a recently reported peptide‐based PET tracer that targets tumoral c‐Met relying on the chelation capabilities of DOTA that was conjugated on the peptide for gallium‐68 chelation. As frequently observed in other cancers, the upregulation of the c‐Met in renal cell carcinoma (RCC) is associated with the overall survival in the metastatic stage (mRCC) of the disease, and the staging of the receptor expression could improve the patient management (i.e., therapy with c‐Met tyrosine kinase inhibitors). Mittlmeier et al.^48^ reported a human study of ^68^Ga‐EMP‐100 in mRCC patients with the aim to evaluate the uptake in primary lesion and in metastases of RCC. Twelve patients with advanced metastatic RCC underwent PET imaging with the novel reported probe and the biodistribution in normal organs and in the lesion were studied. Eighty‐seven tumor lesions were analyzed and 68 were recognized as c‐Met‐positive. Comparing the different analyzed sites, the highest uptake was reported in tumor burden at the primary site followed by bone, lymph node, and visceral metastases. All the other c‐Met negative lesions were heterogeneously distributed intra‐ and interindividually and mostly present as lung and liver metastases. ^68^Ga‐EMP‐100 was physiologically accumulated in the urinary bladder content and kidneys, and only moderate to low uptake was measured in the liver, spleen, pancreas, and intestine. The clinical success of the reported study warrants further research studies investigating the clinical use of ^68^Ga‐EMP‐100 and other similar probes as a biomarker in mRCC patients.

## 5 PET PROBES BASED ON SMALL MOLECULES

**Figure 5:**
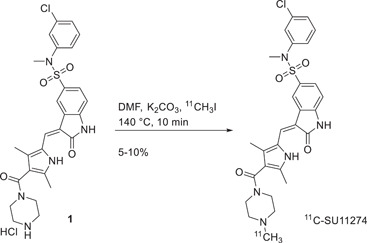
Labeling of SU11274 with ^11^C

HGF, antibodies, and peptide‐based PET probes all have in common that their c‐Met binding sites are located in the extracellular portion of the protein; however, the catalytic activity of c‐Met, that triggers the signal transduction, is activated in its intracellular domain. Thus, small molecules imaging agents that bind the receptor within its intracellular catalytic domain could provide useful knowledge about the activation status of the receptor. Also, small molecules probes, thanks to their low molecular weight and size, are highly permeable in tissue and potentially in cells, and easily cleared. The first study of such compounds for c‐Met was reported by Wu et al.^49^ that produced ^11^C‐SU11274. The designed probe is based on SU11274 is a c‐Met tyrosine kinases inhibitor that was then labeled with carbon‐11 generating ^11^C‐SU11274. The molecule was radiolabeled in a single‐step reaction starting from synthesized molecule **1** (Figure 5). The two molecules (SU11274 and ^11^C‐SU11274) have the same structure, so the specificity and the binding affinity of the probe are preserved. The imaging properties were evaluated by micro‐PET in mice bearing H1975 (c‐Met positive human NSCLC) and mice bearing H520 (c‐Met negative human NSCLC). The measured tumor uptake was significantly higher for H1975 mice than H520; in particular, the one of ^11^C‐SU11274 in c‐Met positive animal model reached its maximum at 80 min postinjection and was constantly higher than the c‐Met negative animal, confirming the usefulness of ^11^C‐SU11274 as a radioactive tracer for detection of the activation of c‐Met by PET imaging.

**Figure 6:**
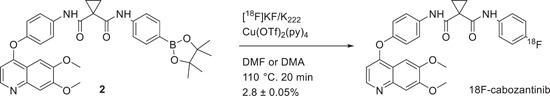
Labeling of cabozantinib with ^18^F

Cabozantinib is another c‐Met kinase inhibitor that was FDA‐approved for the treatment of different types of cancer (advanced RCC and medullary thyroid cancer). The radiosynthesis of ^18^F‐cabozantinib was reported by Lien et al.^50^ starting from a boronic acid derivative **2** (Figure 6). ^11^C‐labeling could also be feasible considering a demethylated quinoline ring precursor and [^11^C]CH_3_I, however, the longer half‐life radioisotope ^18^F (110 vs. 20 min) was chosen over ^11^C for optimal clinical applications and availability. Due to the electron‐donating features of the amide group the SNAr reaction for the introduction of ^18^F would have been impractical, resulting in a multistep reaction starting from ^18^F‐dinitrobenzene, consecutive reduction to aniline, and then formation of the amide bond.^71^ By exploiting a reported approach for the production of ^18^F‐labeled molecules based on the use of boronic acid pinacol esters in copper‐catalyzed reactions with ^18^F‐fluoride they synthesize the ^18^F marked cabozantinib in about 2.8% yield and with a molar activity of 17 ± 8 GBq/μmol. The precursor **2** was produced in four‐step reactions. The method produced only ^18^F‐cabozantinib as a radioactive species and only impurities due to hydrolysis of **2** were identified (reduced to only 0.17% with respect to the final product). A total of ~280 MBq of ^18^F‐cabozantinib was isolated, despite the yield, satisfactory for applications in small animal PET studies that were however not reported in the study.

**Figure 7:**
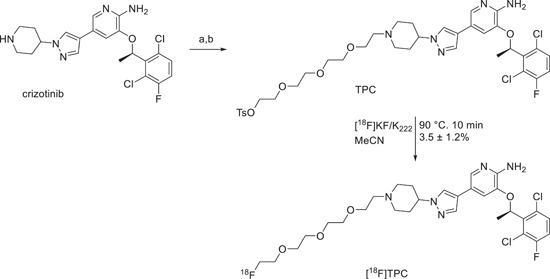
Synthesis and labeling of TCP with ^18^F. (A) Tetraethylene glycol di‐p‐tosylate, cesium carbonate, DMF, room temperature, 2 days, 20%; (B) tetra‐n‐butylammonium fluoride (2 ml, 1 M in THF), room temperature, 36%

More recently another kinase inhibitor‐based PET probe was reported by Lin et al.^51^ The reported probe was designed based on the structure of crizotinib, an FDA‐approved c‐Met kinase inhibitor for the treatment of NSCLC but also in clinical trials for neuroblastoma, anaplastic large cell lymphoma, and other advanced solid tumors. The structure of crizotinib was modified with a polyethylene glycol chain accordingly with the x‐ray cocrystal structure of the targeted protein to not to affect the binding of crizotinib with the receptor. The resulting molecule was called TPC and was used as a precursor for ^18^F‐labeling (Figure 7). TPC was easily labeled with ^18^F exploiting the tosyl group of the PEG‐4 chain bonded to the nitrogen 1 of the piperidine ring as the leaving group, in anhydrous MeCN at 90°C in 10 min to produce the targeted ^18^F‐FPC with a radiochemical yield of 3.5%. The stability of the molecule was proved at 37°C for 6 h. A fast blood clearance was showed in biodistribution studies (4.77 ± 0.07% ID/g at 1 h postinjection), while the uptake in kidney and liver was 29.40 ± 1.77% ID/g and 30.61 ± 1.77% ID/g after 1 h injection, respectively. The metabolism of the molecule was through the hepatobiliary pathway as proved by an increasing ID/g in the large intestine (4.85 ± 2.10% ID/g at 2 h postinjection to 10.12 ± 5.44% ID/g at 6 h postinjection). Optimal uptake over time was measured for cancer tissue that reached 13.68 ± 1.96% ID/g at 1 h postinjection, 6.02 ± 2.83% ID/g at 4 h postinjection, with an optimal tumor to lung ratio of about 3.5 at 2–4 h postinjection. Imaging of xenograft mice H1993 tumor (c‐Met positive NSCLC) or A549 tumor was then performed with ^18^F‐FPC at 4 h postinjection. The radioactivity in H1993 tumor was higher than that in A549, and the uptake of H1993 tumor can be repressed by high doses of crizotinib, confirming ^18^F‐FPC as an optimal candidate for PET scansions of c‐Met‐positive NSCLC tumor.

More generic small‐molecule PET tracers could also be useful for monitoring c‐Met activity in cancer and treatment efficacy. For example, Tseng et al.^72^ showed the possibility of ^18^F‐FDG (fluorodeoxyglucose) to track c‐Met inhibition by CE‐355621 in the glioblastoma U87MG. Cullinane et al.^73^ described that crizotinib inhibition of c‐Met could be tracked in GTL‐16 gastric cancer xenografts and U87MG using ^18^F‐FLT (fluorothymidine), whereas ^18^F‐FDG can only be used in GTL‐16. The two tracers ^18^F‐FDG and ^18^F‐FLT were also useful for prompt monitoring of the effect of BAY 853474 in cancer.^74^ When small molecules probes are considered, the final molecules derived from the labeling precursor should ideally have the same structure of an already known c‐Met small‐molecule binders to preserve the specificity. Moreover, the half‐life of the covalently bonded radioisotope must be long enough to ensure good quality images and enough time between the synthesis/purification of the probe and the injection.

## 6 CONCLUSIONS AND PERSPECTIVE

The frequency of c‐Met receptor misregulation in cancer diseases and its known influence on the pathology evolution make this membrane protein an interesting molecular target for the design of novel diagnostic and therapeutic pharmaceuticals in several cancer types. The already approved c‐Met inhibitor drugs and other Phases II and III clinical studies have shown promising results with a remarkable benefit for the patients in different types of cancers.^75^ In this study, we have reviewed all the c‐Met PET tracers developed up to now, from HGF‐like, to mAbs, peptides, and small molecules, with each substrate type bearing unique advantages as well as limitations. Unfortunately, only one of the reported tracers (^68^Ga‑EMP‑100, Table 1) has been studied in a clinical trial with PET application and research should focus on the translation of more probes in the clinic. While zirconium‐based antibodies have demonstrated good results in preclinical studies, other probes with smaller structures like peptides and anticalines have also showed good in vivo properties, with the advantages of being compatible with more popular short half‐life radionuclides, for example, ^18^F.^76^ On the other hand, smaller organic molecules have better pharmacokinetics but they are cleared fast from the body which translates in nonoptimal probes for delayed over time imaging. A good compromise in our opinion is the family of the peptide‐based tracers as exemplified by the clinical application (via fluorescence detection and recently via PET with ^68^Ga‐EMP‐100, Table 1) of GE‐137 resulting in promising impact for detection of malignant polyps and RCC. The main advantages of the peptide‐based targeting moieties are that they are cheaper than antibodies and can be easily chemically modified to be radiolabeled with virtually any radionuclide making them attractive for clinical use.^77^ However, PET agents based on small organic compound tyrosine‐kinase inhibitors have the advantage to directly bind the intracellular binding site of the receptor and be more precise in quantifying the activation of the receptor.

Despite the scarcity of the clinical data for c‐Met targeting PET probes, and only the recent first in human application of such tools, some general advantages and drawbacks for this pharmaceutical class can be summarized as commonly reported for the application of other PET radiopharmaceuticals and should be taken into account when considering the future application of c‐Met PET probes. For instance, the application of PET probes other than the already discussed diagnosis and therapy monitoring is the use of PET and PET/CT for tumor ablation and guided biopsies.^78^ In this field the major advantages of using a PET‐guided clinical tumor ablation are to combine anatomical imaging with functional imaging. Moreover, the prolonged half‐life of the used nuclides warrants continual localization of the tissue target during the clinical procedure.^79^ The capability to accurately target and ablate large tumors and the possibility of estimating the success at the end of the surgery are other advantages of assisted clinical ablation by PET imaging.^79^ But in this application the main disadvantages are related to the radiation exposure for the patients together with the clinical operators. With respect to diagnosis, although PET imaging is not a primary choice for staging early stage breast cancer, it has a key role in systemic staging for this and other types of solid tumors and in comparison with MRI, PET/CT has been proved to have more specificity for breast cancer detection but less sensitivity.^80^, ^81^ Overall, PET/MRI overperforms the detection of primary breast cancer when compared to PET/CT, but the improvement is limited if in comparison with MRI only. Moreover, ^18^F‐FDG can localize early‐stage bone marrow metastases before they appear on bone scintigraphy or CT with PET scansion.^82^ Predicting the therapeutic response and optimizing it accordingly is a further application of PET imaging for breast and other solid cancers. Unfortunately, as stated in the ongoing clinical guidelines, PET imaging is not beneficial neither for early‐stage breast cancer (i.e., large screening of population) in the absence of symptoms nor for the classification of clinical stages of breast cancer.^81^ However, PET imaging coupled with CT and MRI adds additional information to standard diagnostic imaging techniques in the detection process of nodal disease and other metastases. Nevertheless, further optimization is still mandatory to reach advanced performances and efficiency, and novel technological advanced PET probes such as those reported in this review will have a primary role in this stage. Finally, when working with PET probes other common limitations could be: renal excretion which may limit the detection in retroperitoneum and pelvis area, short half‐life nuclides (e.g.,^11^C) resulting in cyclotron produced limiting availability, still limited role in primary staging and first diagnosis, target (e.g., c‐Met, PSMA, or other receptors) high dependency, high bone marrow uptake, which may limit the detection of bone metastases, and significant liver and/or other tissue uptake, which may reduce the detection of metastatic disease in those areas.

The merging of accurate imaging radiotracers with PET will allow noninvasive and real‐time diagnosis of specific cancer types with accurate picture of tumors and metastases and thus precise staging of the disease. Moreover, the assessment of the studied protein expression, the uptake kinetics, and the pretherapeutic dosimetry may grant a better selection of the treatments as well as monitoring the patient response to the applied therapy and anticipate detection of recurring disease resulting in personalized medicine and radiotheranostic (e.g., probe based on ^67/68^Ga).^83^

The recent progress in the research field of c‐Met based PET imaging here reviewed are highly encouraging, even if the optimization of a PET probe for clinical purposes still faces challenges, suggesting that this family of tracers could be useful as a diagnostic tool for patients selection and for therapeutic applications in the near future.

## Data Availability

Data sharing not applicable to this article as no data sets were generated or analyzed during the current study.
